# “*…When it came to sensitive information*,* we made edits*,* and we took it back”*: qualitatively exploring the role responsibilities taken on by Canadians who crowdfund on behalf of someone else from a privacy perspective

**DOI:** 10.1186/s12910-025-01312-3

**Published:** 2025-10-28

**Authors:** Benjamin Lartey Nii Badu, Valorie A. Crooks, Jeremy Snyder

**Affiliations:** 1 Department of Geography, Simon Fraser University, Burnaby, BC Canada; 2 Faculty of Health Sciences, Simon Fraser University, Burnaby, BC Canada

**Keywords:** Medical crowdfunding, Ethics, Privacy, GoFundMe, Canadians, Healthcare services, Qualitative research

## Abstract

**Background:**

Medical crowdfunding, a type of donation-based crowdfunding, is gaining prominence and enabling people to gather funds for medical treatments, surgeries, and other health needs. While this practice may democratize access to health care, it also raises ethical concerns, including breaching individuals’ privacy. Despite these concerns, little consideration has been given specifically to the privacy-related issues that emerge when people crowdfund on behalf of others’ health-related financial needs.

**Methods:**

A study was undertaken to qualitatively explore the roles and associated role responsibilities of Canadians who crowdfund on behalf of others for their health needs. Twelve interviews were conducted with participants who had posted campaigns on the GoFundMe platform between January and December 2023. Interviews were transcribed, coded, and analyzed thematically.

**Findings:**

Three key roles that had important privacy dimensions were identified: managing initial content, navigating informational considerations, and facilitating ongoing connections. Campaigners typically collaborated with recipients to craft compelling narratives, seek consent for sharing personal information, and provide regular updates to maintain donor engagement. Balancing campaign transparency with recipients’ privacy concerns was crucial in the crowdfunding process.

**Conclusion:**

Campaigners play pivotal roles in medical crowdfunding when doing so on behalf of funding recipients, including balancing the need for fundraising with the protection of recipients’ privacy. Clear guidelines are needed to support campaigners in navigating the ethical complexities that emerge. Further research is needed to address existing knowledge gaps and enhance the ethical integrity of crowdfunding practices.

**Supplementary Information:**

The online version contains supplementary material available at 10.1186/s12910-025-01312-3.

## Background

Medical crowdfunding enables people to gather funds to cover expenses such as preventative care treatments, surgeries, and other health needs through crowd sourcing [1]. Through online platforms like GoFundMe, people grappling with health-related costs can extend their reach for financial help to broad audiences in the hope of receiving substantial financial supports from family members, friends, and even unknown or distant donors [2–4]. Medical crowdfunding has gained momentum not only in countries like the United States, where care costs are known to be high, but also in places with ‘universal’ healthcare systems such as Canada, where essential medical treatment has no cost at the point of care [5, 6]. For Canadians, medical crowdfunding serves as an additional avenue to fund extra treatments, specialized care, or unforeseen expenses linked to illness or injury that are not covered publicly [7]. It is thus not surprising that GoFundMe has raised substantial funds for Canadian campaigns, including for health needs [8, 9]. In addition, the use of social media platforms such as Facebook and Instagram has made it even easier for people facing health challenges to amplify their stories by sharing crowdfunding campaigns to solicit financial support [10–12]. Some have argued that medical crowdfunding has democratized access to health services, especially among marginalized populations, by lessening or doing away with personal financial barriers [13–15].

Despite the altruism enabled by medical crowdfunding [16], serious ethical concerns have emerged around matters related to fraud, equity, and autonomy [17]. Concerns also exist among ethicists and others regarding the potential for crowdfunding to threaten the privacy of those featured in campaigns [18]. Privacy can be conceptualized in many ways, with a common element being having control over the disclosure of personal information or knowledge about oneself to others [19, 20], which is what we are focused on in the current analysis. Publicly sharing personal details that would not typically be revealed to people beyond one’s immediate social circle without permission may constitute a breach of privacy [17]. In medical crowdfunding campaigns, achieving success often depends on effectively sharing detailed information about someone’s health status with a wider audience, with the aim of fostering empathy among potential donors [7]. In addition, crowdfunding platforms allow campaigners to use photos and videos to share imagery related to their lives, health status, and financial needs [21]. Campaigners are also urged to provide frequent updates on the advancement of their campaign and health situations to sustain donor involvement and inspire further donations [22, 23]. All of these instances present opportunities for campaigners to trade privacy protection for the potential to receive donations from others. The ethical issue of privacy protection may very well be further complicated when campaigners initiate and manage fundraising efforts on behalf of others, which is the situation we explore in the current analysis. It has been hypothesized that in some cases these campaigners might lack the authorization to disclose personal information about the intended recipient [7], though research knowledge is lacking.

When crowdfunding campaigns breach privacy, significant implications can emerge for those involved. These can include the erosion of trust, compromise of integrity, infringement upon recipients’ rights, and loss of confidentiality [24]. Moreover, risks such as unintended exposure can potentially lead to public judgment and even generate adverse consequences that can threaten family and friend relationships [25]. These risks are particularly salient for those crowdfunding to support medical costs related to stigmatized needs, such as access to abortion care or trans-affirming treatment [26–28]. Requests to fund such medical care can influence the public perception of those about whom campaign content is written and may expose them to negative responses from others [29]. While the implications of privacy breeches such as these have been identified, little consideration has been given to if and how those campaigning on behalf of recipients take action to avoid such outcomes given the roles they play in creating the public-facing content.

Although ethicists and others have deepened our understanding of how and why privacy is an ethical issue in the practice of medical crowdfunding [7, 17], there is a lack of primary research that documents how these concerns are navigated by campaigners. Further to this, much of the crowdfunding literature treats all campaigners as the same. Yet, as we pointed out above, those campaigning on behalf of others’ health needs may have particular privacy considerations to address given the sensitivities involved in sharing personal health information. The current analysis takes an important step towards addressing this knowledge gap by presenting the findings of interviews conducted with Canadians who created health-related crowdfunding campaigns on behalf of a fundraising recipient. More specifically, we explore the key roles that these campaigners take on, and their associated responsibilities in light of these roles, all of which have important privacy considerations. We believe that such insight provides important nuance not only to the existing literature on the ethics of medical crowdfunding, but also to calls put forth to better inform crowdfunders about the privacy risks associated with this practice and to create opportunities to enhance informed consent in the practice of crowdfunding [7, 17].

## Methods

This exploratory qualitative analysis contributes to a larger study seeking to comparatively understand how decision-making regarding the disclosure of personal information is conceptualized by Canadians participating in charitable crowdfunding campaigning. The larger study has involved conducting in-depth interviews with Canadians who are crowdfunding for themselves and those who are doing so on behalf of other recipients, with a focus on fundraising for health needs. The current analysis focuses on the latter interview dataset, allowing for a deep consideration of the practices that inform how crowdfunding on behalf of others may, or may not, raise privacy concerns. We focused on health-related campaigns because of their commonness and also because the nature of the information disclosed is more likely to be understood as private (e.g., diagnostic status, health updates, etc.) relative to, for example, campaigns to cover personal travel or wedding costs [17].

Upon receiving ethics approval, we used the GoFundMe crowdfunding platform to identify potential participants. This crowdfunding platform was selected to support recruitment because of its popularity and use among Canadians [30]. Between January, 2023 and December, 2023, we used a webcrawler to extract information on newly posted campaigns and searched these records for ones that met our inclusion criteria. Given the exploratory nature of our study and the lack of population-level data regarding involvement in crowdfunding, the cut-off for data collection was guided by a temporal end point that reflected our available time and research funding resources rather than a target number of participants. We specifically sought campaigns on GoFundMe that were posted in Canada by an adult (i.e., 18 + years of age) who was not the intended funding recipient to support costs brought on by health needs. For campaigns that met these criteria, we searched the name of the campaigner to see if an e-mail address or social media profile that we could contact could be identified, which was a time intensive process. In cases where contact information could be identified, e-mails or messages were sent to campaigners to solicit their consent for participation in an interview should they be age 18 or older. In total, we identified 176 publicly accessible profiles of people who fulfilled the inclusion criteria on Facebook to whom we sent direct messages. Because our messages were unsolicited and not coming from people in the known social networks of those with whom we were in touch with, they landed in a secondary ‘message requests’ inbox that significantly lessened their visibility. Seven people we were in touch with through Facebook agreed to participate in an interview. We found publicly accessible e-mail addresses for another 62 campaigners who met our inclusion criteria to whom we reached out. Five agreed to participate in an interview.

Campaigners who met the inclusion criteria and expressed interest in participating were provided with study information and consent details prior to the interview. Participants were also asked to provide and authenticate links to their crowdfunding campaign websites. This enabled us to confirm that potential participants had indeed organized a crowdfunding campaign. One-on-one in-depth interviews were conducted using Zoom video call in English and started by capturing participants’ verbal consent. Interviews often lasted between 45 and 90 min and were conducted by the first author. A semi-structured interview guide was used, which covered matters relating to: decision to crowdfund, results of crowdfunding campaign initiatives, privacy in everyday life, and privacy when crowdfunding. Interviews were digitally recorded with participant consent. After the interviews, participants received CAD$25 e-gift cards to acknowledge their contribution to the study.

Digital interview recordings were transcribed verbatim in preparation for coding and thematic analysis. Thematic analysis involves identifying, analyzing, and interpreting patterns of meaning within qualitative data [31]. As a first step, all authors independently reviewed four transcripts to deepen familiarity with the dataset and identify potential themes. Following this, the team met to discuss analytic directions, using investigator triangulation to confirm the scope and scale of dominant themes. Concepts informing these dominant themes were then identified, which were used to form the development of a coding scheme that also drew from the study objective and interview prompts. Manual coding in a word processing program ensued using the scheme, which was done by the first author. Upon completion of the coding, data relating to the current analysis were extracted and again shared with the team. Following independent review, another team meeting was held to confirm interpretation and the analytic focus. This included discussion of the various responsibilities identified through independent review and a collaborative working of these data to identify connections among responsibilities that, when grouped together, reflected larger roles. This process of investigator triangulation led to complete consensus among the team regarding the presence of the roles and responsibilities focused herein across the dataset as well as the scope of each. Consistent with thematic analysis, literature relating to the analytic focus reported herein was reviewed at this point to support opportunities for contextualizing the findings and their novelty. Overall, the analytic process led to the identification of three primary roles taken on by those crowdfunding for someone else’s health needs, which we discuss in detail in the section that follows. Throughout this discussion we integrate verbatim quotes from participants to support trustworthiness and authenticity of interpretation.

### Findings

Twelve Canadians who had crowdfunded on behalf of a fundraising recipient agreed to participate in an interview for this exploratory study. All participants resided in the Canadian provinces of British Columbia (67%) and Ontario (33%). Eleven identified as women and one as a man, with ages spanning from 22 to 64 years. The requested amounts from the campaigns using GoFundMe ranged from CAD$5,000 to $100,000, with four participants meeting their campaign targets. The highest amount raised in a single campaign was CAD$37,843. Participants’ education levels varied from high school to a graduate degree, and family annual incomes ranged from CAD$30,000 to CAD$160,000. Campaigners sought funding for various needs, including: covering medical expenses such as cancer treatment and surgeries; medical travel within Canada; home repairs and maintenance to support needed accommodations; as well as supplementing income for recipients unable to work due to their health. Eleven participants collaborated with campaign recipients from the planning stages, discussing campaign goals and plans for sharing personal information. The remaining participant initiated a campaign as a gesture of goodwill, later informing the recipient’s family. Participants’ close relationships with recipients, such as being family members (*n* = 3) or close friends (*n* = 9), facilitated collaboration and helped campaigners act in the recipients’ best interests while respecting their privacy preferences. They were motivated to fundraise on behalf of recipients out of a desire to support these friends and family in challenging times, noting that in many instances recipients’ health status was a barrier to self-fundraising due to limited energy or capacity. In a few instances, participants indicated that they had expertise related to communications, fundraising, or marketing that best positioned them to manage the campaign.

Through thematic analysis we identified three key roles, along with their associated responsibilities, that crowdfunders took on when campaigning on behalf of someone else that have implicit and explicit privacy dimensions. We conceptualized role responsibilities as the ethical duties and obligations that campaigners undertook to protect recipients’ privacy and uphold the integrity, transparency, and fairness of the crowdfunding process while also acting to support a positive outcome (i.e., obtaining campaign donations). These roles, which are explored in this section, were: managing initial content; navigating informational considerations; and facilitating ongoing connections. All participants reported undertaking each these roles when crowdfunding on behalf of a campaign recipient. The ‘managing initial content’ role related to tasks undertaken to create and post a campaign. The ‘navigating informational considerations’ role pertained to tasks associated with managing the balance between addressing privacy concerns and creating appealing campaigns. Finally, the ‘facilitating ongoing connections’ role related to the tasks associated with updating campaign contents with narratives to connect people and identifying key donors and audiences across diverse networks and encouraging sharing. In the sub-sections that follow, we expand on these roles through discussing the primary responsibilities associated with each that were identified through thematic analysis of the twelve interviews conducted with participants. Though we discuss these roles separately, we acknowledge there are interconnections and explore some in the discussion section.

### Managing initial content

All participants highlighted the importance of creating and posting relatable crowdfunding campaign narratives to garner both financial and emotional support from others. The process of crafting appealing narratives when creating a campaign involved the careful selection of information including photos, videos, and images. This role responsibility was mostly undertaken in collaboration with the campaign recipient and sometimes also close others, such as spouses and family members. Participants explained that this collaborative process was reassuring as it ensured that campaign narratives accurately represented the views and personal stories of recipients, including personal health details. A 23-year-old campaigner explained: “*…I drafted a big story*,* and then information we didn’t want to share got edited out.”* Taking leadership of managing initial campaign content illustrated the nuanced nature of engagement with recipients from the outset, with numerous participants emphasizing the importance of respecting the autonomy and preferences of recipients and obtaining their consent to post information when campaigning on behalf of someone else’s health needs.

An important role responsibility associated with managing the initial campaign content was the identification of financial needs and setting a campaign goal. Part of this involved determining the extent of the financial request. For example, if the recipient had medical care needs but was also unable work due do their health, participants grappled with determining if the financial request would focus only on the direct medical care costs or if it would also integrate the wider financial gaps brought on by not working. There was no single strategy most commonly used among participants to determine the scope of the financial request to be made from the outset of the campaign. What was common, however, was some engagement with the recipient, which may also have involved others in their networks. Participants commonly had close personal relationships with recipients, such as *“…I was raising money for my best friend’s family…”* or *“…it was for my friend and her husband…*”, and these connections drove participants’ desires to present a fulsome and well justified campaign financial goal from the outset in the hopes of success.

### Navigating informational considerations

Seeking consent and approval in the context of sharing personal information on behalf of recipients was a critical role responsibility discussed by participants. This was rooted in participants’ shared recognition of the rights of recipients to control their personal information and make informed decisions about how it was shared and presented. As one participant explained, “*…I would hope that anyone who crowdfunds on another person’s behalf has their permission and their consent*.” Recognizing this, participants highlighted making needed adjustments and edits throughout the lifecycle of the campaign (i.e., initial content, campaign updates, shares on social media, etc.) to align with the recipient’s approach to protecting their privacy. This was reflected in the following viewpoint shared by a 43-year-old campaigner: “*…when it came to sensitive information*,* we made edits*,* and we took it back*.” A common motivation for crowdfunding on behalf of a recipient was to lessen any additional burden placed on the recipient due to their health and medical care contexts. It was thus not surprising that the extent to which detailed and/or ongoing approvals were sought for information updates throughout the campaign’s lifecycle varied across participants given recipients’ fluctuating abilities and availabilities.

Participants touched on the need to balance privacy concerns against creating appealing, information rich campaigns as an important matter. This role responsibility was influenced by both the need to garner support from donors and safeguard recipients’ private information. The emphasis on “*…giving enough information that they feel like they have empathy for the family*,* but not so much that people feel like they have a reason to investigate these people’s lives*” highlighted the importance of striking a balance between transparency and discretion. Recognizing this need for balance, some participants reported focusing on broadly discussing health information rather than getting into diagnostic details, for example. Overall, participants emphasized the need to honor privacy-driven requests about information sharing made by recipients over the need to create content that would inspire donations. By respecting these boundaries, they could preserve trust in their interactions with recipients.

### Facilitating ongoing connections

Participants highlighted their role responsibility to regularly update campaigns to keep potential or existing donors engaged with, connected to, and informed about the recipient and their story. They understood that keeping donors informed and involved was important not only for retaining interest, but also for developing stronger relationships that might have led them to share campaigns via their social networks. A participant explained that “*…every time we went to update it*,* it was just so that the more people understood what he was going through*,* the more that people felt familiar with him and what was going on*.” Participants also recognized the importance of expressing gratitude towards donors through updates: “*As a thank you*,* we wanted to update them on his progress and status*.” There was, however, strong acknowledgement by all participants that updates contributed to donations. As one participant explained, “*…before I had a big description*,* I wasn’t getting as many donations. Once I added a big description and put updates and shared information about the family*,* donations did improve*.” As noted in this quote, while updates presented opportunities to facilitate connection with and inform the decision-making of donors, they also served as content where privacy trade-offs needed to be carefully navigated.

According to participants, identifying target donors and audiences across social media networks and encouraging sharing was strategically indispensable. Participants discussed engaging with both familiar and new donors and audiences to amplify the potential success of the campaign. This sentiment was shared by a 49-year-old campaigner who explained: “*…I changed my [social media] settings to public…I wanted it to be [reaching] maybe a stranger that felt moved by the story and had the means to donate*.” Another explained that “*…I was very intentional about it. I reached out to people I knew who were active on social media*.” A small number of participants maintained a more intimate and personalized approach to outreach, “*…we just kept it to our networks. I wanted it to be kind of personal*.” Overall, participants’ experiences emphasized that such flexibility and intentionality when crowdfunding for someone else’s health needs was integral to effectively engaging with various audiences. Effective audience engagement, in turn, was often necessary to achieve the fundraising goal and balanced with effectively facilitating connection with the recipient.

## Discussion

Thematic analysis of the twelve interviews has shown that organizing a crowdfunding campaign for someone else’s health needs includes three distinct roles: managing initial content, navigating informational considerations, and facilitating ongoing connections. A number of responsibilities are associated with each of these roles, which are synthesized in Table 1. These roles have many important interconnections that are underscored in the findings shared above. For example, role responsibilities relating to navigating informational considerations were enacted in relation to managing initial content and facilitating ongoing connections. Decisions made related to managing initial content with regard to the amount of information shared about the recipient shaped the depth of ongoing connections to donors and others interacting with the campaign that was possible. In the remainder of this section, we contextualize the analytic findings in relation to the existing literature on crowdfunding in addition to considering directions for future research and study strengths and limitations.

**Table 1:** Roles and their associated role responsibilities

Roles	Role Responsibilities
Managing Initial Content	• Creating and posting crowdfunding campaign narratives on a selected platform • Identifying financial needs and setting a campaign goal
Navigating Informational Considerations	• Engaging with recipients to gain approval for content shared in campaigns, including in updates • Balancing privacy concerns against creating an appealing campaign
Facilitating Ongoing Connections	• Updating campaign contents with narratives to connect people • Identifying key donors and audiences across diverse networks to encouraging sharing

This analysis has shown how at their core, medical crowdfunding campaigns created on behalf of a known recipient rely on careful planning and collaboration. Campaigners collaborate with recipients to plan and craft narratives, select photos and visuals to articulate the campaign’s purpose – all of which are viewed by campaigners to be very important components of crowdfunding campaigns [32, 33]. Identifying recipients’ financial needs and setting campaign goals are another key responsibility that involves careful planning and collaboration. Concurrently, as recipients may choose to forgo privacy in order to maximize fundraising, campaigners facilitate achieving informed consent from recipients regarding the administration of the campaign. Ultimately, navigating informational considerations requires campaigners to take on a fiduciary role where they seek input from and then act in the best interests of recipients [34]. This further requires striking a careful balance between protecting recipients’ privacy and providing sufficient information to inspire donations.

While the navigation of the balance between protecting privacy and campaign success has been previously discussed in the ethical literature on medical crowdfunding more generally [5, 7, 17, 35], the current study has provided illustrations of when and how this occurs in contexts where people are campaigning on behalf of others. Similarly, in facilitating ongoing connections, a collaborative approach was common to carefully plan and identify target audiences or donors and limit the disclosure of sensitive information during updates and sharing across diverse social media networks. It is important to point out that not all recipients know when crowdfunding campaigns are posted on their behalf, which has been underscored by the literature examining fraud in relation to this practice [5, 36]. Although an inclusion criterion in the current study was not ensuring that recipients knew about campaigns that were posted on their behalf, it was the case that in all instances campaigners collaborated with known recipients who were closely positioned in their family or friend networks. Further research should meaningfully explore differences in how the fiduciary relationship between campaigners and recipients is enacted when recipients know about campaigns versus when they do not.

It is noteworthy that the roles identified in this analysis have not been extensively explored in the existing literature on donation-based crowdfunding. However, we acknowledge that existing research has elucidated various responsibilities undertaken by campaigners more generally during crowdfunding. For example, prior studies have shown how the success of crowdfunding campaigns often depends on creating appealing narratives and posting images that resonates with potential donors [32, 33, 37–42]. In the current analysis, such activity is conceptualized as a role responsibility related to the managing initial content role undertaken by those campaigning on behalf of someone else. Zheng and Jiang [39], reviewed patients’ stories, emotional appeals, campaign clarity, and narrative structure and their positive impacts in medical crowdfunding campaigns [39]. Liu et al. [41] analyzed how approaches such as the use of direct gaze in campaign images and storytelling approaches influence the success of crowdfunding for medical purposes [41, 43]. These prior studies align with our findings to further confirm that the responsibility of creating appealing narratives and posting imagery is crucial in the medical crowdfunding landscape. This very responsibility has been critically examined in the ethics literature in particular because, in many ways, the trade-off between sharing personal details and images to gain donations threatens privacy in potentially harmful ways [44]. What the current analysis has demonstrated is an awareness of this potential harm by those crowdfunding on behalf of a recipient and a desire to mitigate it through collaboration.

Crowdfunders campaigning on behalf of others must navigate complex ethical issues, including privacy protection and respecting recipients’ autonomy, while also seeking to raise donations and help the recipient [5, 7, 17, 44]. Platforms such as GoFundMe do not provide tailored campaign submission guidelines and or content development tools that are specific for those campaigning on behalf of others. Meanwhile, this analysis has shown that there are particular roles and role responsibilities taken on by these campaigners that may benefit from particular consideration. Training support for these campaigners along with privacy guidelines would be highly useful, as would educational initiatives that raise awareness about privacy risks and develop informational tools targeting both campaign creators and donors. As noted by others, these informational interventions should emphasize explicit consent and appropriate disclosure of information [45, 46]. The current analysis serves as a reminder to consider the distinct informational needs of particularly situated campaigner groups in the development of such tools.

The findings of this exploratory study point to several important directions for future research. First, much research on crowdfunding explores this practice through analyzing data gathered from campaigns [7, 8, 30, 47]. While such analyses have garnered incredibly important insights about equity and ethical issues associated with this practice and its outcomes, there is much to learn from directly engaging with people involved in crowdfunding as campaigners, recipients, and donors, among others. The nuanced roles and role responsibilities identified in the current analysis serve as a case in point. There is much opportunity for future research to further engage directly with involved groups. Second, this analysis provides an important illustration of the value of considering differences within crowdfunding stakeholder groups. A core premise of this analysis is that crowdfunding on behalf of someone else is fundamentally different from crowdfunding for oneself, yet much existing research on crowdfunding does not differentiate between types of campaigners. Most differentiation that does exist focuses on campaign types, such as the particular needs of those crowdfunding for cancer care [48, 49], paediatric neurology [50], or Lyme disease diagnosis and treatment [8]. Third, future research could build on this analysis by exploring the related role responsibilities taken on by crowdfunding campaign funding recipients. This could include, for example, examining if they undertake any continued measures to safeguard privacy in relation to decisions regarding how, where, and when to spend campaign proceeds. Finally, another critical issue in need of further research consideration relates to the ethical dilemmas inherent in medical crowdfunding. Despite the existence of literature on this topic (e.g. [17, 36, 51, 52]) we point out the necessity to further address the unforeseen complexity of ethical concerns when crowdfunding for others’ health needs. Such research can ultimately inform the creation of clearer guidelines and/or regulatory measures in relation to crowdfunding based on trust, integrity, and the preservation of recipients’ rights and privacy [5, 7, 44].

### Limitations

We acknowledge the following limitations in our study. Firstly, the use of Zoom video calls for interviews rather than face-to-face interviews limited our ability to observe non-verbal signals including body language and gestures. This may have negatively impacted rapport building and the sharing of detailed experiences [52]. However, the use of Zoom allowed us to interact with participants from anywhere in Canada, which is a benefit that outweighed any potential limitations associated with using this medium. Another limitation is that, due to the lack of population-level data availability, we do not know how representative the participants we spoke with are of those who crowdfund on behalf of others. For example, significantly more people who identified as women participated in interviews than men. This may be due to women’s stronger presence on social media sites [53], or may be a reflection of who crowdfunds for others. Women often take on caregiving roles, which may extend to managing health care costs and coordinating crowdfunding campaigns on behalf of others [54]. As this study is qualitative, we have not sought to have a representative sample and have instead provided context about participants that can facilitate transferability, which is an indicator of rigour in qualitative research.

## Conclusions

This exploratory qualitative study has shown the various roles and associated role responsibilities campaigners undertake when crowdfunding to meet others’ health needs. Through thematic analysis, we identified three key roles they play: managing initial content, navigating informational considerations, and facilitating ongoing connections. These roles intersect and collectively show the importance of collaboration in respecting recipients’ privacy preferences when crowdfunding on behalf of someone. The scope and scale of these roles also highlight the complexity associated with navigating privacy considerations. This complexity calls for careful planning, particularly if fund recipients’ autonomy and privacy-related requests are to be honoured. These findings meaningfully contribute to the existing literature on the ethics of medical crowdfunding [55], particularly by providing insights into the nuanced dynamics of campaigners’ roles and responsibilities and highlighting the benefit of giving dedicated research attention to particular campaigner groups. Significant knowledge gaps remain about crowdfunding that, if appropriately addressed, can support the development of informational tools and resources that can guide campaigners, recipients, donors and other stakeholders involved to enhance the ethical nature of this practice and ultimately the equity of outcomes.

## Supplementary Information

Supplementary Material 1.

## Data Availability

To maintain participants’ privacy and anonymity, interview transcripts from this study are not publicly available.
