# Supplementary material for: “…When it came to sensitive information, we made edits, and we took it back”: qualitatively exploring the role responsibilities taken on by Canadians who crowdfund on behalf of someone else from a privacy perspective
Source: BMC Med Ethics. 2025 Oct 28;26:149. doi: 10.1186/s12910-025-01312-3 (PMC12560443; doi:10.1186/s12910-025-01312-3)
Supplement: Supplementary file 1 — Supplementary Material 1. [file 12910_2025_1312_MOESM1_ESM.docx]

**Interview Guide**

- **About You**
  - In what province do you live?
  - What gender to you identify as?
  - What is your age range?
    - 18-29
    - 30-39
    - 40-49
    - 50-59
    - 60-69
    - 70 or older
  - What is your household income range?
    - 0-$24,999
    - $25,000-$49,999
    - $50,000-$74,999
    - $75,000-$99,999
    - $100,000-$124,999
    - $125,000-$149,000
    - $150,000-$200,000
    - $200,000-$249,000
    - $250,000+
  - What is your educational background?
    - Some secondary
    - Completed secondary
    - Some post-secondary
    - Completed post-secondary
    - Some graduate
    - Completed graduate
- **Deciding to Crowdfund**
  - What were you trying to raise money for?
  - Why did you choose crowdfunding as a way to raise this money?
  - Did you try any other means of raising this money, either before or after the crowdfunding campaign?
  - How did you become aware of crowdfunding as an option?
  - Had you ever donated to a crowdfunding campaign before starting your own?
  - Did you try to share your campaign as widely as possible or keep it within friends and family? Why?
  - Did you regularly update your campaign with additional information? Why or why not?
  - How did you determine the financial goal of the campaign?
- **Results of the Campaign**
  - Did you meet your crowdfunding goal?
  - What factors do you think contributed to getting people to donate? What factors do you think held people back from donating?
    - Did your campaign receive any wider attention, such as media coverage?
  - Is there anything you would do differently in terms of running your crowdfunding campaign?

*I’d like to ask you some questions about how you manage your private information in your life generally and how you did so in your crowdfunding campaign. Private information can be many different things, like your age, photos of yourself, past experiences, and information about your family members. Many people have very different approaches to privacy and care more or less about what is private and what is public. What we have in mind is the difference between aspects of yourself and your loved ones that you are comfortable having open to the public compared to those aspects you want to have control over who knows or sees.*

- **Privacy in Everyday Life**
  - In general, how open are you about sharing details about your private life with friends and family?
  - Do you use social media? If so, do you share personal details? Images? Videos?
    - Do your decisions to share personal details vary between social media types (e.g., Instagram vs. Facebook), and why?
  - What personal information are you more reluctant to share with others and what are you more open about normally?
    - Your location (e.g., tracked via phone)?
    - Your address?
    - Your phone number?
    - Your birth date, including year?
    - Your credit scores?
    - Your income?
    - What internet sites you visit?
    - Your purchasing history?
    - Your email correspondence?
    - Your medical history?
    - Your employment history?
  - How do you decide what personal information to share with others? Do you have specific rules like no pictures of your kids online? No birthdates? Nothing without permission? Anything goes?
- **Privacy in Crowdfunding**
  - Given what we’ve already talked about in relation to privacy, in general what kinds of privacy issues do you think exist in relation to crowdfunding?
    - Were you concerned about any of these issues in relation to your campaign?
      - If so, did these concerns change throughout your experience of campaigning?
  - How did you determine what personal details you would post in the campaign description?
    - Do you remember actively deciding not to include details in the campaign because you were concerned that they were too revealing? Please tell me about this.
  - How did you decide what photos and videos you would post in the campaign?
    - Do you remember actively deciding not to include images or videos in the campaign because you were concerned that they were too revealing? Please tell me about this.
  - Were there particular topics that felt more sensitive or private than others? Medical? Family? Financial?
  - Did you feel like you had complete control over what personal details were disclosed in your campaign?
    - Was there any pressure to share as much detail as possible in order to meet your goals? If so, how did that make you feel? How did you respond to this pressure?
    - Did you feel like you were fully in control over what you shared? If so, why is that?
- **Privacy when Raising Money for Others**
  - How did you come to organize the campaign?
    - Did the campaign recipient consent to the campaign? Provide information for you to post? Provide photos to post?
  - Were you in regular communication with the campaign recipient (or their guardian) regarding the campaign?
    - About what?
  - Did you and the beneficiary discuss any issues that you felt were relevant to privacy, and what were they?
  - How was the recipient involved in deciding how personal information was shared in the campaign or did you need to make some of these decisions on your own? Were they most involved, equally involved, or less involved?
    - Did their involvement help with or complicate how decisions over information sharing and privacy were made? How so?
- **Reflecting Back**
  - Is there anything you would do differently about crowdfunding in general and sharing personal information in your campaign specifically?
  - What advice around privacy would you give to another person considering crowdfunding?
  - Who do you think has a responsibility to make any changes or give any advice around privacy in crowdfunding?

Thank you for your time. Is there anything that we didn’t cover that you think I should know about your experience with crowdfunding, especially as it relates to privacy?
